# Genome-Wide CRISPR/Cas9 Screening Identifies Modulators of THZ1 Response in Acute Myeloid Leukemia

**DOI:** 10.3390/biomedicines14051113

**Published:** 2026-05-14

**Authors:** Weidong Ding, Xiaoya Yun, Yifan Liu, Hui Liu

**Affiliations:** 1 Department of Hematology, Beijing Hospital, National Center for Gerontology, National Clinical Research Center for Gerontology, The Key Laboratory of Geriatrics of NHC, Institute of Geriatric Medicine, Chinese Academy of Medical Sciences & Peking Union Medical College, No. 1 Dahua Road, Dongdan, Dongcheng, District, Beijing 100005, China; b2023021018@pumc.edu.cn (W.D.); hlm_ssby@126.com (X.Y.); liuyifan079@126.com (Y.L.); 2 Graduate School of Peking Union Medical College, Chinese Academy of Medical Sciences, Beijing 100730, China

**Keywords:** acute myeloid leukemia, CDK7, THZ1, *TP53*, *MYC*, *E2F*, CRISPR/Cas9

## Abstract

**Background/Objectives:** Acute myeloid leukemia (AML) remains in need of more broadly effective therapeutic strategies. THZ1, a covalent CDK7 inhibitor, has shown anti-leukemic activity in AML, but the mechanism underlying its response remains incompletely defined. This study aimed to identify key modulators and related transcriptional programs involved in THZ1 response in AML. **Methods:** We combined a genome-wide CRISPR/Cas9 screen with functional validation and transcriptomic analyses. **Results:** *TP53* was the top positively selected gene in the CRISPR screen in MOLM-13 cells. THZ1 induced p53 accumulation, and pharmacologic activation of the p53 pathway with idasanutlin further enhanced the early anti-leukemic response to THZ1 at 24 h. *TP53* loss reduced the sensitivity of AML cells to THZ1-induced apoptosis, but did not abolish THZ1 responsiveness. Transcriptomic analyses showed that *TP53* loss substantially reshaped the transcriptional state, whereas *MYC* and *E2F* target programs remained the most consistent pathways linked to THZ1 response. THZ1 also continued to suppress these programs in *TP53*-knockout MOLM-13 cells and *TP53*-mutant THP-1 cells. **Conclusions:** Overall, *TP53* contributes to the anti-leukemic response to THZ1 but does not determine it, and THZ1 continues to suppress *MYC* and *E2F* target programs after *TP53* loss.

## 1. Introduction

Acute myeloid leukemia (AML) is a highly heterogeneous hematologic malignancy. Its development is associated with complex genomic abnormalities and diverse mutational profiles, which influence clinical outcome and treatment response [1]. For many years, the treatment landscape of AML has changed only modestly. Despite continued efforts to optimize intensive chemotherapy, improve combination regimens, and introduce new agents, overall therapeutic progress has remained limited [2,3]. In recent years, advances in AML genomics and molecular classification have promoted the clinical development of targeted therapies against BCL-2, FLT3, and IDH1/2. These agents have expanded treatment options and improved outcomes in selected patient subsets [4,5]. However, responses remain heterogeneous, and primary or acquired resistance continues to limit the durability of remission. More broadly effective therapeutic strategies therefore remain needed in AML.

One potential strategy is to target transcriptional dependencies that support AML cell survival. AML cells rely not only on mutation-specific oncogenic signaling but also on dysregulated transcriptional programs that sustain leukemic cell growth, preserve stem-like states, and impair myeloid differentiation [6,7,8]. These transcriptional states may persist or be remodeled under therapeutic pressure and have been linked to chemotherapy resistance and relapse in AML [9,10]. These findings provide a rationale for targeting core transcriptional regulatory machinery as a complementary approach to standard cytotoxic and targeted therapies in AML. In this context, CDK7 is of particular interest because it links cell cycle control with RNA polymerase II-mediated transcription. It forms the CDK-activating kinase complex with Cyclin H and MAT1. This complex activates multiple cell cycle-related CDKs through T-loop phosphorylation, thereby promoting cell cycle progression. CDK7 is also a core component of the general transcription factor TFIIH. During transcription initiation, it phosphorylates the C-terminal domain of RNA polymerase II and promotes transcriptional initiation [11]. THZ1 is a covalent inhibitor of CDK7 and has shown potent antitumor activity in multiple solid tumors, including pancreatic ductal adenocarcinoma, gastrointestinal stromal tumors, urothelial carcinoma, and breast cancer [12,13,14,15,16]. THZ1 has also demonstrated strong antitumor effects in hematologic malignancies, including peripheral T-cell lymphoma, B-cell acute lymphoblastic leukemia, and multiple myeloma [17,18,19,20]. In AML, the t(8;21) subtype is highly sensitive to THZ1, and brief exposure is sufficient to induce marked apoptosis [21]. Our previous study further showed that THZ1 induces apoptosis in multiple AML model systems and acts synergistically with azacitidine in vitro and in vivo [22]. Together, these findings support the translational relevance of THZ1-mediated CDK7 inhibition as a transcription-directed therapeutic strategy in AML. However, the genetic modulators and transcriptional programs associated with AML cell responses to THZ1-mediated CDK7 inhibition remain incompletely defined.

To address this question, we performed a genome-wide CRISPR/Cas9 screen to identify genetic modulators and related pathways that influence THZ1 response in AML cells. We then integrated functional experiments and transcriptomic analyses to define the role of *TP53* and transcriptional programs in this response.

## 2. Methods

### 2.1. Chemicals and Reagents

THZ1 (Catalog No. T3664) and idasanutlin (Catalog No. T6254) were purchased from TargetMol (Boston, MA, USA).

### 2.2. Cell Culture

MOLM-13 and THP-1 cell lines were purchased from Cobioer Biosciences Co., Ltd. (Nanjing, China). MOLM-13 is a widely used AML cell line derived from a patient with FAB-M5a acute monocytic leukemia and harbors FLT3-ITD, a clinically relevant genetic lesion in AML. Both cell lines were cultured in RPMI-1640 medium (GIBCO, Thermo Fisher Scientific, Waltham, MA, USA) supplemented with 10% fetal bovine serum (FBS, GIBCO) and 1% penicillin–streptomycin (P/S, GIBCO). Cells were maintained at 37 °C in a humidified incubator with 5% CO_2_.

### 2.3. Genome-Wide CRISPR/Cas9 Knockout Screen

**Scheme 1:**
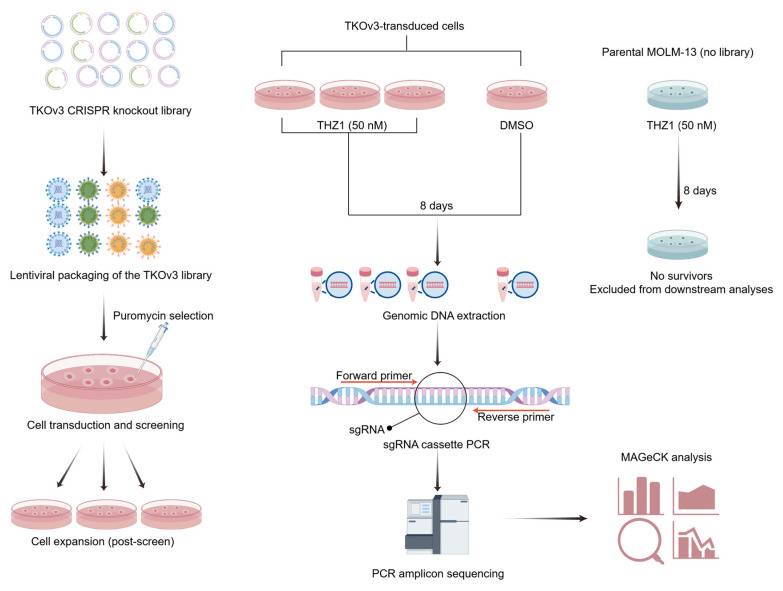
Workflow of the genome-wide CRISPR/Cas9 screen. The pooled TKOv3 sgRNA library was packaged into lentivirus and transduced into MOLM-13 cells. Following 7 days of puromycin selection and expansion while maintaining ≥500 × sgRNA coverage, cells were divided into three THZ1 (50 nM, 8 days) replicates and one DMSO control. Parental MOLM-13 lacking the library failed to survive THZ1 and were excluded. Genomic DNA from TKOv3-transduced cells was collected, sgRNA cassettes were PCR-amplified and sequenced on an Illumina platform, and read counts were summarized with MAGeCK for gene-level enrichment and depletion analyses.

A genome-wide CRISPR/Cas9 knockout screen was performed in MOLM-13 cells using the Cas9-expressing lentiviral vector lentiCRISPR v2 (Addgene, Watertown, MA, USA; #90294) and the TKOv3 sgRNA library (Scheme 1) [23,24]. The library comprises 70,948 sgRNAs targeting 18,053 human genes, with approximately four sgRNAs per gene. Cells were infected at a multiplicity of infection of 0.3 to 0.5 to favor single-copy integration. After 48 h, cells were selected with 1 μg/mL puromycin for 7 days. Surviving cells were expanded and divided into four groups. Three groups were treated with 50 nM THZ1, and one group was treated with DMSO. Treatment was continued for 8 days. Each group contained approximately 4 × 10^7^ cells, providing about 500-fold coverage of the library. Genomic DNA was then extracted, and sgRNA cassettes were amplified by PCR for high-throughput sequencing. MAGeCK (Version. 0.5.9.4) was used to identify significantly enriched and depleted genes and pathways [25].

### 2.4. CRISPR/Cas9-Mediated TP53 Knockout

Lentiviral particles carrying a *TP53*-targeting sgRNA were generated by co-transfecting 293T cells at approximately 80% confluence with pLenti-U6-gRNA-Cas9-P2A-EGFP-Puro&Zeocin (Beyotime Biotechnology, Shanghai, China; #D8303) encoding the sgRNA sequence ACCAGCAGCTCCTACACCGG, psPAX2 (Addgene, #12260), and pMD2.G (Addgene, #12259) at a ratio of 4:3:1 using Lipofectamine 3000 (Thermo Fisher Scientific; #L3000015). Viral supernatants were collected 48 h after transfection, centrifuged at 4000 rpm for 10 min, and passed through a 0.45 μm filter. MOLM-13 cells were infected with the viral suspension and selected with 1 μg/mL puromycin for 7 days beginning 48 h after infection. EGFP-positive cells were isolated by flow cytometric sorting and cultured for 3 weeks to derive single-cell clones. *TP53* knockout was confirmed by Western blotting, and clones with no detectable p53 protein expression were selected as p53-null *TP53*-knockout MOLM-13 cells for subsequent experiments.

### 2.5. Western Blot

Whole-cell lysates were prepared using RIPA lysis buffer supplemented with PMSF and protease inhibitor cocktail. Protein concentrations were determined using a BCA Protein Assay Kit (Epizyme Biomedical Technology, Shanghai, China; #ZJ102). Equal amounts of protein were separated by SDS-PAGE and transferred onto 0.45 μm PVDF membranes. Membranes were blocked with 5% nonfat milk in TBST and incubated with an anti-p53 primary antibody (Cell Signaling Technology, Danvers, MA, USA; #2524) or anti-GAPDH primary antibody (Abcam, Cambridge, UK; #ab181602) overnight at 4 °C, followed by HRP-conjugated secondary antibodies. Signals were detected using ECL reagent (Meilunbio, Dalian, China; #MA0186).

### 2.6. Cell Viability, Drug Sensitivity, and Combination Analysis

Cell viability was measured using a Cell Counting Kit-8 (CCK-8; Dojindo Laboratories, Kumamoto, Japan; #CK04). MOLM-13 cells were seeded in 96-well plates at 1 × 10^4^ cells per well and treated with single agents or drug combinations at the indicated concentrations. At the indicated time points, CCK-8 solution was added to each well at a 1:10 dilution, followed by incubation at 37 °C for 4 h. Absorbance at 450 nm was measured using a microplate reader (Thermo Fisher Scientific; #1410101). Cell viability was normalized to untreated controls. Dose–response curves were fitted by nonlinear regression using GraphPad Prism 8.0.2, and IC50 values were calculated.

For pharmacologic activation of the p53 pathway, MOLM-13 cells were treated with idasanutlin, a selective MDM2 antagonist, either alone or in combination with THZ1. For the combination assay, cells were treated with a dose matrix of THZ1 (0–24 nM) and idasanutlin (0–640 nM). Normalized cell viability data were analyzed using SynergyFinder Plus (Version. 07.09.2024). Drug interactions were evaluated using the Highest Single Agent (HSA) and Loewe models. For each model, synergy scores were calculated across the entire dose matrix, and the corresponding matrix-level *p* values were reported. Synergy scores greater than 10 were considered synergistic, scores between −10 and 10 were considered additive, and scores less than −10 were considered antagonistic [26].

### 2.7. Apoptosis Assay

Apoptosis was measured using an Annexin V-PE/7-AAD Apoptosis Detection Kit (Multi Sciences, Hangzhou, China; #AP104). MOLM-13 cells were seeded in 6-well plates at 1 × 10^6^ cells/mL and treated with the indicated drugs or the corresponding control condition until the indicated time points. After treatment, cells were collected, washed with pre-cold PBS, and resuspended in 1× binding buffer. Approximately 5 × 10^5^ cells were stained with 5 μL Annexin V-PE and 10 μL 7-AAD for 10 min at room temperature in the dark, followed by immediate flow-cytometric analysis. Unstained and single-stained samples were used for compensation and gating. At least 1 × 10^4^ valid events were acquired for each sample. Cell debris was excluded based on FSC/SSC plots, and cell populations were quantified using Annexin V-PE and 7-AAD two-parameter plots. Early apoptotic cells were defined as Annexin V+/7-AAD−, and late apoptotic cells were defined as Annexin V+/7-AAD+. The residual viable-cell fraction was defined as Annexin V−/7-AAD− and normalized to the 0 h baseline within each genotype for quantitative comparison.

### 2.8. Quantitative Real-Time PCR (RT-qPCR)

Total RNA was extracted using RNAiso reagent (Takara Bio Inc., Kusatsu, Shiga, Japan; #9108) according to the manufacturer’s instructions. cDNA was synthesized from total RNA using the PrimeScript RT reagent Kit (Takara, #RR092A). Quantitative PCR was performed using TB Green Premix Ex Taq II (Takara, #RR420A) on a real-time PCR system. *GAPDH* was used as the internal control, and relative gene expression was calculated using the 2^−ΔΔCt^ method. The primer sequences used in this study are listed in Supplementary Table S1.

### 2.9. RNA Sequencing and Bioinformatic Analysis

RNA-seq data for THP-1 cells treated with 50 nM THZ1 for 24 h were obtained from our previous study (GSA-Human HRA002252). Public RNA-seq data were retrieved from the GEO database under accession GSE131592, from which the *TP53*-knockout MOLM-13 dataset was used for analysis [27]. Differential expression analysis was performed using DESeq2 in R (Version. 4.3.0). Gene set enrichment analysis (GSEA; v4.3.3) was performed using the Molecular Signatures Database (MSigDB) Hallmark collection (release 2024.1; h.all.v2024.1.Hs.symbols), and ssGSEA was performed using the GSVA package (Version. 2.2.0) in R [28,29].

### 2.10. Statistical Analysis

Statistical analyses were performed using GraphPad Prism 8.0.2 and R. For differential expression analysis, genes with *p* < 0.05 and |log2FC| > 0.585 were considered differentially expressed. For pathway-level analyses, pathways with *p* < 0.05 were considered significant. For cell viability, apoptosis, qPCR, and immunoblot quantification involving comparisons between two groups, statistical significance was assessed using an unpaired Student’s *t* test. For p53 protein expression across multiple time points, normality was assessed using the Shapiro–Wilk test. No significant deviation from normality was detected; therefore, statistical significance was assessed using ordinary one-way ANOVA followed by Dunnett’s multiple comparisons test, with the 0 h group as the control. A *p* value of less than 0.05 was considered statistically significant.

## 3. Results

### 3.1. Genome-Wide CRISPR/Cas9 Screening Identifies Genes and Pathways Associated with the THZ1 Response

**Figure 1:**
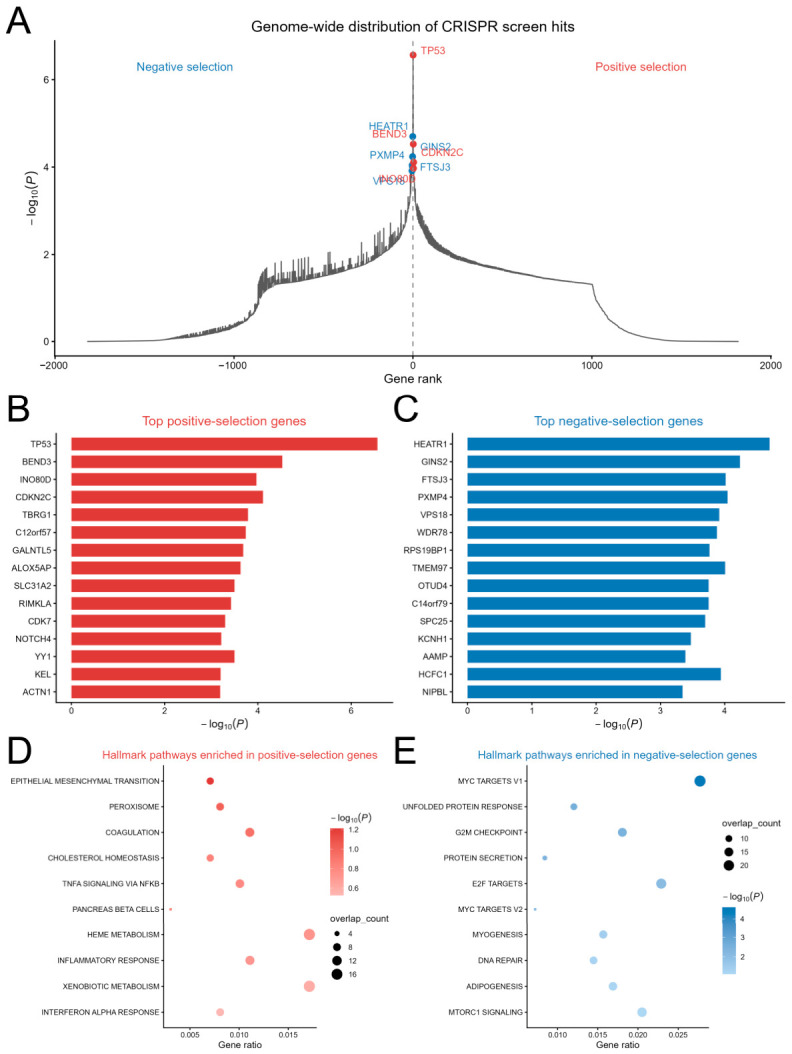
Genome-wide CRISPR/Cas9 screening identifies modulators of the THZ1 response in MOLM-13 cells. (**A**) Genome-wide distribution of CRISPR screen hits in MOLM-13 cells under THZ1 selection. Positively selected genes represent candidates whose loss conferred relative resistance to THZ1, whereas negatively selected genes represent candidates whose loss enhanced THZ1 sensitivity. Representative genes are labeled. (**B**) Top positively selected genes ranked by screening significance. (**C**) Top negatively selected genes ranked by screening significance. (**D**) Hallmark pathway enrichment analysis of positively selected genes. (**E**) Hallmark pathway enrichment analysis of negatively selected genes.

To define modulators of the THZ1 response in AML cells, we performed a genome-wide CRISPR/Cas9 knockout screen in MOLM-13 cells under THZ1 selection pressure and analyzed gene-level effects using MAGeCK. The overall distribution of screen hits is shown in Figure 1A. Positively selected genes represented candidates whose loss conferred relative resistance to THZ1, whereas negatively selected genes represented candidates whose loss enhanced THZ1 sensitivity. Among the positively selected genes, *TP53* ranked first (Figure 1B), indicating that *TP53* loss provided a relative survival or growth advantage under THZ1 selection pressure. In this context, positive selection refers to sgRNA enrichment after THZ1 treatment, consistent with a resistance-associated effect of gene loss. In contrast, *HEATR1* was the top negatively selected gene (Figure 1C). These results indicate that loss of distinct genes has different effects on THZ1 responsiveness.

To further characterize the screen at the pathway level, we performed Hallmark enrichment analysis using the positively and negatively selected gene sets. Positively selected genes were associated with epithelial–mesenchymal transition, peroxisome, coagulation, and inflammatory signaling programs (Figure 1D). Negatively selected genes were associated with *MYC* and *E2F* target programs, G2M checkpoint, unfolded protein response, DNA repair, adipogenesis, and mTORC1 signaling (Figure 1E).

### 3.2. THZ1 Induces p53 Accumulation and Enhances Anti-Leukemic Activity When Combined with Idasanutlin

**Figure 2:**
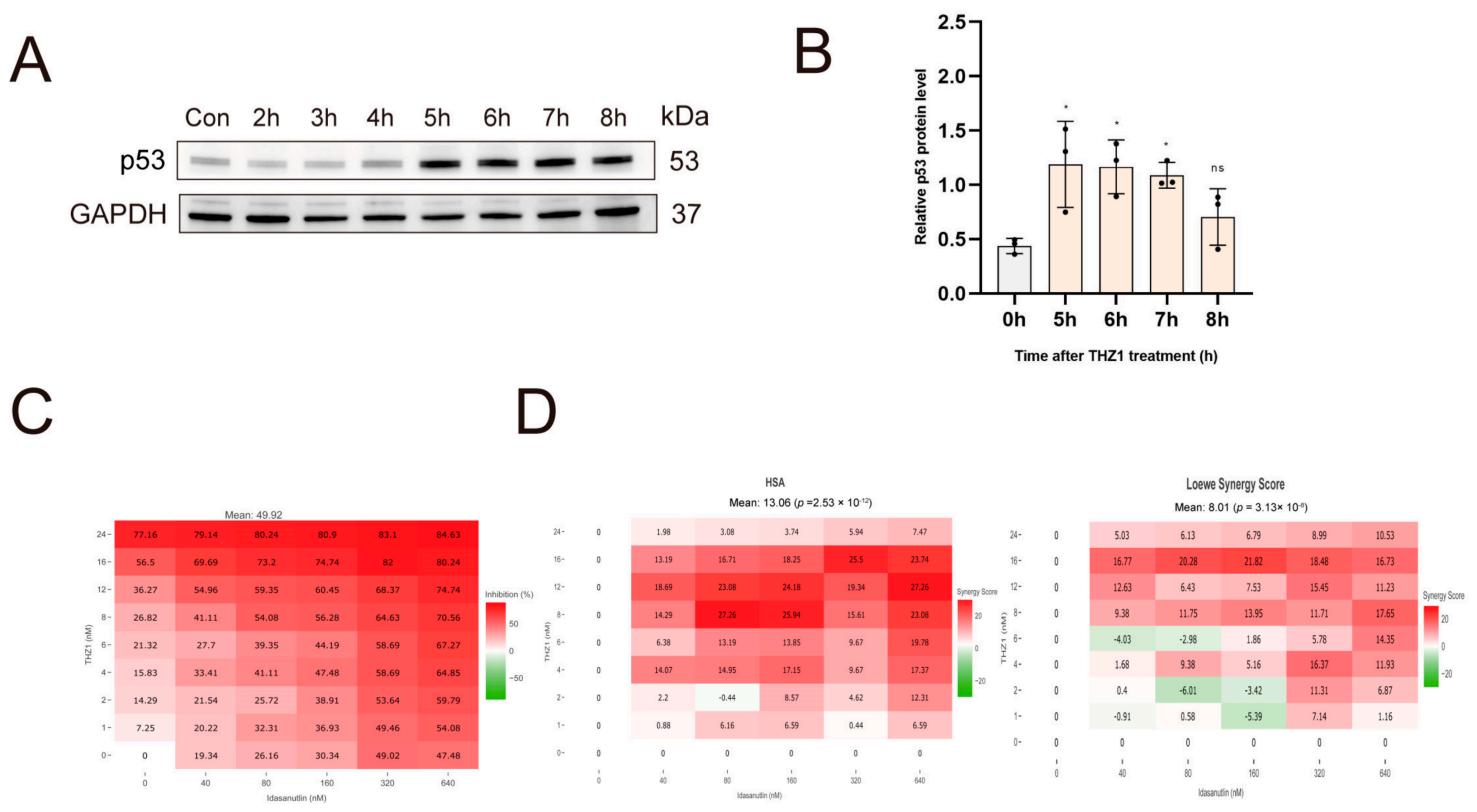
THZ1 induces p53 accumulation, and idasanutlin enhances the early anti-leukemic response to THZ1 at 24 h. (**A**) Representative immunoblot showing p53 protein levels in MOLM-13 cells treated with THZ1 for the indicated times. GAPDH was used as a loading control. (**B**) Quantification of p53 protein levels at time points included across all independent repeats. Protein expression was normalized to GAPDH and is presented relative to the 0 h group. Data are presented as mean ± SD. ns, not significant; * *p* < 0.05. (**C**) Heatmap showing growth inhibition in MOLM-13 cells after 24 h of treatment with THZ1 and idasanutlin at the indicated concentrations. Numbers in each cell represent the percentage of growth inhibition. (**D**) Synergy heatmaps for the combination of THZ1 and idasanutlin in MOLM-13 cells, analyzed using the Highest Single Agent (HSA) and Loewe models. Positive scores indicate synergistic interaction. For the HSA model, the mean synergy score was 13.06 (*p* = 2.53 × 10^−12^), and the maximum synergy score was 27.26. For the Loewe model, the mean synergy score was 8.01 (*p* = 3.13 × 10^−8^), and the maximum synergy score was 21.82.

Because *TP53* was the top positively selected gene in the CRISPR/Cas9 screen and has clear biological and clinical relevance in AML, we selected it for further analysis. We first examined whether THZ1 was associated with activation of the p53 pathway. Immunoblotting and densitometric analysis showed that THZ1 increased p53 protein expression in MOLM-13 cells, with significant increases observed at 5 h (mean relative level 1.189, *p* = 0.0128), 6 h (1.166, *p* = 0.0155), and 7 h (1.088, *p* = 0.0289) compared with 0 h (0.438) (Figure 2A,B).

To determine whether this response had functional relevance, we next tested whether pharmacologic activation of the p53 pathway could modulate THZ1 sensitivity. Idasanutlin is a selective MDM2 antagonist that blocks the MDM2-p53 interaction and thereby stabilizes wild-type p53. After 24 h of combined treatment, THZ1 plus idasanutlin produced stronger growth inhibition than either agent alone across the tested dose matrix, with a mean inhibition of 49.92% and a maximal inhibition of 84.63% (Figure 2C). Synergy analysis using SynergyFinder showed predominantly positive interaction scores. Using the Highest Single Agent (HSA) model, the combination showed a mean synergy score of 13.06 across the dose matrix (*p* = 2.53 × 10^−12^) and a maximal score of 27.26. Additional analysis using the Loewe model showed a mean synergy score of 8.01 across the dose matrix (*p* = 3.13 × 10^−8^) and a maximal score of 21.82 (Figure 2D).

These findings suggest that THZ1 engages a p53-associated response in AML cells, and that pharmacologic activation of the p53 pathway can enhance the early anti-leukemic response to THZ1 at 24 h.

### 3.3. TP53 Loss Attenuates but Does Not Abolish the Anti-Leukemic Activity of THZ1

**Figure 3:**
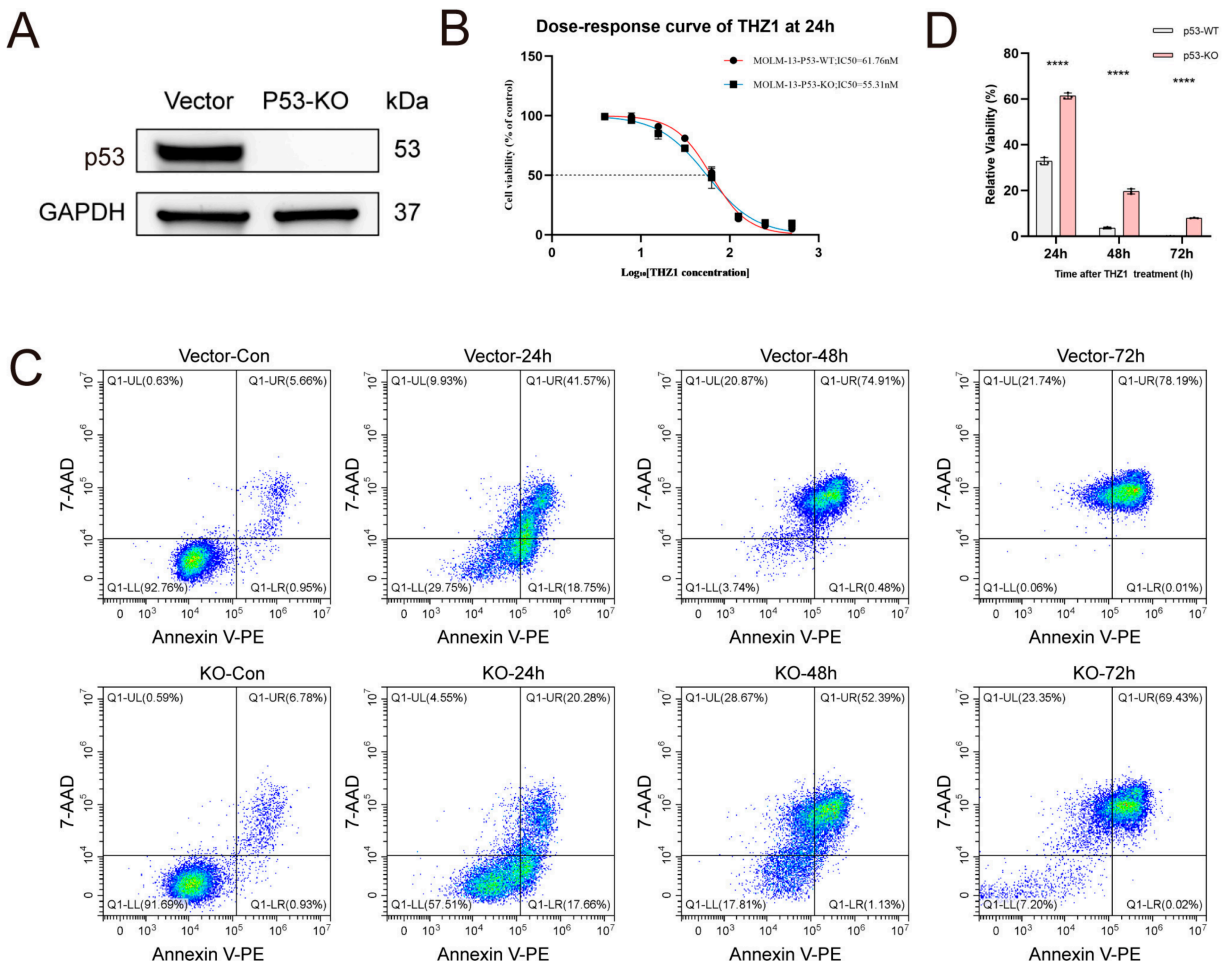
*TP53* loss attenuates but does not abolish the anti-leukemic activity of THZ1. (**A**) Immunoblot analysis confirming p53-null status in *TP53*-knockout MOLM-13 cells. GAPDH was used as the loading control. (**B**) Dose–response curves of THZ1 in vector control and *TP53*-knockout MOLM-13 cells after 24 h of treatment. Cell viability was measured by CCK-8 assay, and IC50 values were calculated for each group. The dashed line indicates 50% cell viability. (**C**) Representative flow-cytometric plots of Annexin V-PE/7-AAD staining in vector control and *TP53*-knockout MOLM-13 cells treated with 50 nM THZ1 for 24, 48, and 72 h. (**D**) Quantification of the residual viable-cell fraction after 50 nM THZ1 treatment for 24, 48, and 72 h. Values were normalized to the 0 h baseline within each genotype. Data are presented as mean ± SD. **** *p* < 0.0001.

To determine whether *TP53* is essential for the anti-leukemic activity of THZ1, we generated *TP53*-knockout MOLM-13 cells and compared their responses with those of vector control cells. Immunoblotting confirmed that *TP53*-knockout MOLM-13 cells were p53-null, with no detectable p53 protein expression (Figure 3A). Dose–response analysis at 24 h showed that THZ1 retained comparable growth-inhibitory activity in both groups, with IC50 values of 61.76 nM in vector control cells and 55.31 nM in *TP53*-knockout cells (Figure 3B).

We next asked whether *TP53* loss affected THZ1-induced apoptosis over time. Flow-cytometric analysis using Annexin V-PE/7-AAD staining showed that THZ1 increased apoptosis in both vector control and *TP53*-knockout cells at 24, 48, and 72 h (Figure 3C,D). At each time point, the normalized residual viable-cell fraction was significantly higher in *TP53*-knockout cells than in vector control cells, with mean values of 61.33% versus 32.81% at 24 h (*p* < 0.0001), 19.57% versus 3.60% at 48 h (*p* < 0.0001), and 7.93% versus 0.06% at 72 h (*p* < 0.0001). These results indicate that, compared with *TP53*-wild-type cells, *TP53* loss reduced the sensitivity of AML cells to THZ1-induced apoptosis. Notably, THZ1 still induced progressive apoptosis in *TP53*-knockout cells over time, with the normalized residual viable-cell fraction decreasing from 61.33% at 24 h to 19.57% at 48 h and 7.93% at 72 h.

Taken together, these findings suggest that *TP53* contributes to the apoptotic response to THZ1, but is not essential for AML cells to respond to THZ1.

### 3.4. THZ1 Retains Its Ability to Suppress MYC and E2F Target Programs After TP53 Loss

**Figure 4:**
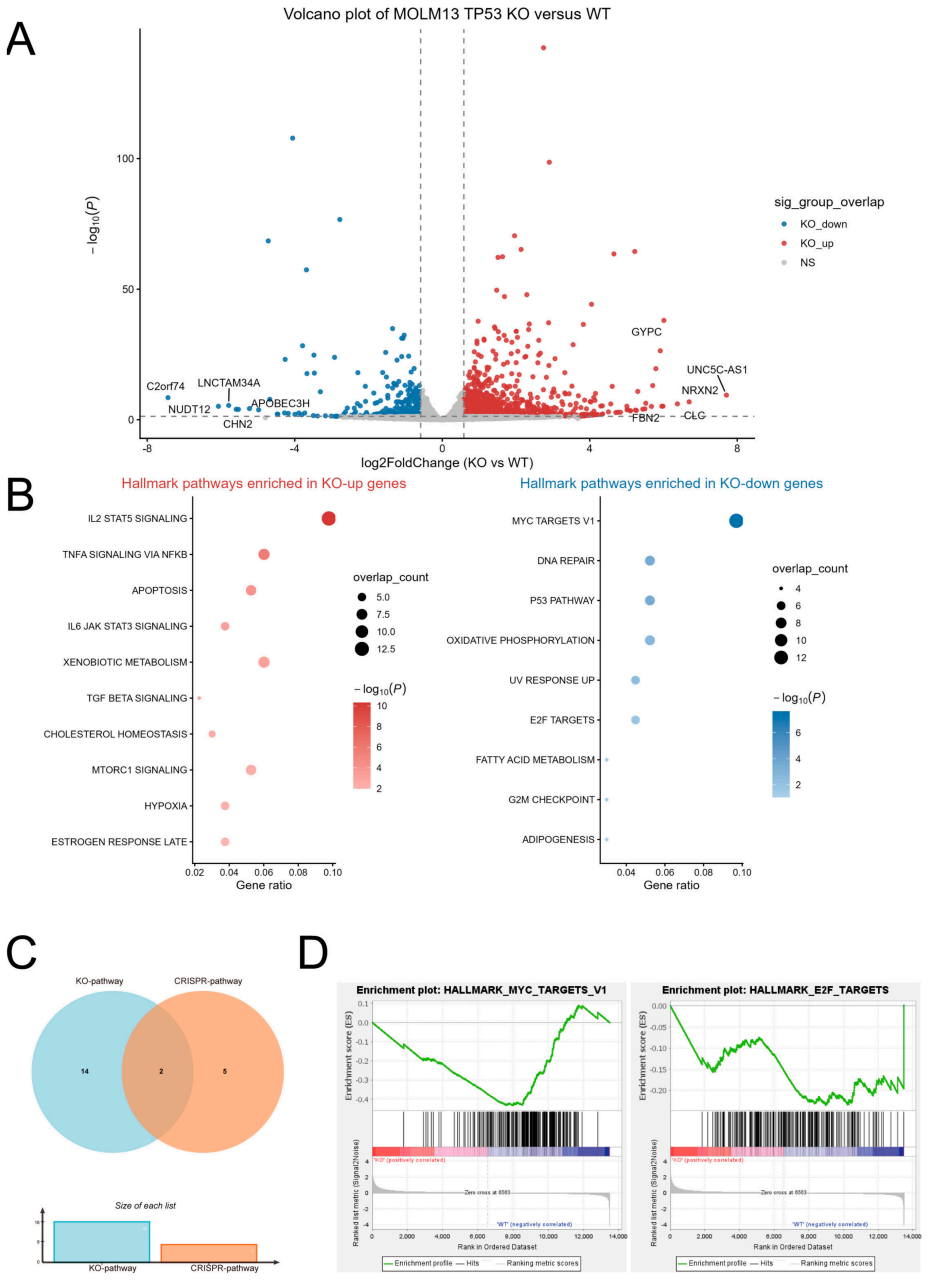
*TP53* loss reshapes the transcriptional state and highlights *MYC* and *E2F* target programs as shared pathways linked to THZ1 response. (**A**) Volcano plot showing differentially expressed genes in *TP53*-knockout MOLM-13 cells relative to control cells. Red dots indicate upregulated genes, blue dots indicate downregulated genes, and gray dots indicate genes without significant change. The dashed vertical lines indicate log2FC = ±0.585, and the dashed horizontal line indicates *p* = 0.05. (**B**) Hallmark pathway enrichment analysis of genes altered after *TP53* knockout. (**C**) Venn diagram showing overlap between pathways associated with *TP53*-knockout transcriptomic changes and pathways identified from the CRISPR screen. *MYC* targets and *E2F* targets were the only shared pathways between the two analyses. (**D**) GSEA plots showing negative enrichment of HALLMARK_MYC_TARGETS_V1 and HALLMARK_E2F_TARGETS in *TP53*-knockout MOLM-13 cells relative to control cells.

To further characterize the transcriptional changes associated with *TP53* loss, we analyzed the transcriptomic profiles of *TP53*-knockout and *TP53*-wild-type MOLM-13 cells (Figure 4A). Hallmark analysis showed that genes upregulated after *TP53* knockout were associated with IL2-STAT5 signaling, TNFA signaling via NFκB, apoptosis, IL6-JAK-STAT3 signaling, and xenobiotic metabolism. By contrast, downregulated genes were enriched in *MYC* and *E2F* target programs, as well as DNA repair, p53 pathway, oxidative phosphorylation, fatty acid metabolism, and G2M checkpoint signatures (Figure 4B).

We next compared the pathway changes associated with *TP53* knockout with the pathway-level dependencies identified by the CRISPR screen. *MYC* and *E2F* target programs were the only pathways shared by both analyses (Figure 4C). GSEA further confirmed negative enrichment of these two programs in *TP53*-knockout cells relative to *TP53*-wild-type cells, with an NES of −1.51 for HALLMARK_MYC_TARGETS_V1 and −0.84 for HALLMARK_E2F_TARGETS (Figure 4D). Thus, although *MYC* and *E2F* target programs were reduced after *TP53* loss, they remained the most consistent pathways linked to THZ1 response across the transcriptomic and CRISPR-based analyses.

**Figure 5:**
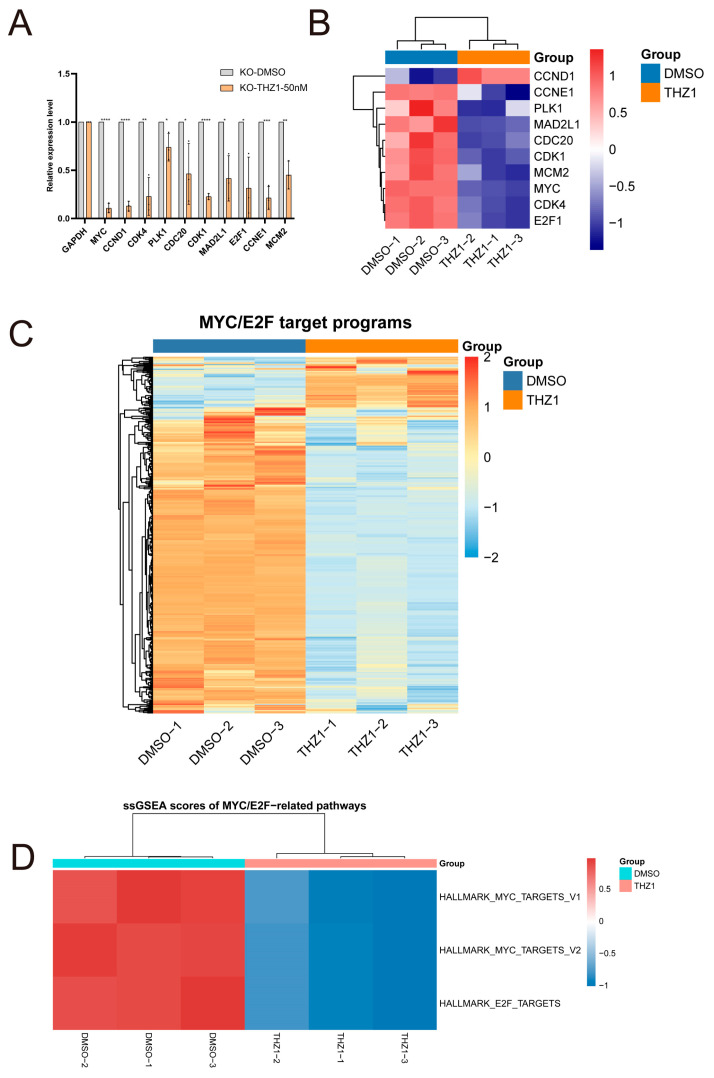
Suppression of *MYC* and *E2F* target programs by THZ1 is retained in *TP53*-knockout MOLM-13 and *TP53*-mutant THP-1 cells. (**A**) qPCR analysis of *MYC* and *E2F* target genes in *TP53*-knockout MOLM-13 cells treated with DMSO or 50 nM THZ1. Data are presented as mean ± SD. * *p* < 0.05, ** *p* < 0.01, *** *p* < 0.001, **** *p* < 0.0001. (**B**) Heatmap showing expression changes of the same *MYC* and *E2F* target genes in THP-1 cells treated with 50 nM THZ1 for 24 h. (**C**) Heatmap showing expression changes of genes from *MYC* and *E2F* target programs in THP-1 cells treated with 50 nM THZ1 for 24 h. (**D**) Heatmap of ssGSEA scores for HALLMARK_MYC_TARGETS_V1, HALLMARK_MYC_TARGETS_V2, and HALLMARK_E2F_TARGETS in THP-1 cells treated with DMSO or 50 nM THZ1 for 24 h.

To determine whether THZ1 could still suppress *MYC* and *E2F* target programs after *TP53* loss, we analyzed *MYC* and *E2F* target genes in *TP53*-knockout MOLM-13 cells. qPCR analysis showed that 50 nM THZ1 significantly reduced the expression of multiple *MYC* and *E2F* target genes, including *MYC*, *CCND1*, *CDK4*, *PLK1*, *CDC20*, *CDK1*, *MAD2L1*, *E2F1*, *CCNE1*, and *MCM2*, and all of these changes were statistically significant (*p* < 0.05) (Figure 5A).

We next examined whether a similar pattern could also be observed in THP-1 cells, which carry mutant *TP53*. In this model, RNA-seq data from THP-1 cells treated with 50 nM THZ1 for 24 h showed coordinated downregulation of the same *MYC* and *E2F* target genes (Figure 5B). Heatmap analysis of genes from HALLMARK_MYC_TARGETS_V1, HALLMARK_MYC_TARGETS_V2, and HALLMARK_E2F_TARGETS further showed broad suppression of *MYC* and *E2F* target programs after 50 nM THZ1 treatment for 24 h (Figure 5C). Consistently, ssGSEA showed reduced activity of these pathways in THZ1-treated cells relative to control cells (Figure 5D). Together, these findings indicate that suppression of *MYC* and *E2F* target programs by THZ1 is retained in *TP53*-knockout MOLM-13 cells and *TP53*-mutant THP-1 cells, supporting the view that this component of THZ1 activity is not fully dependent on intact *TP53* signaling.

## 4. Discussion

In this study, we combined a genome-wide CRISPR/Cas9 screen with functional validation and transcriptomic analyses to investigate the modulators of THZ1 response in AML. *TP53* emerged as the top positively selected gene in the CRISPR screen. We further found that THZ1 induced p53 accumulation and that pharmacologic activation of the p53 pathway with idasanutlin enhanced the anti-leukemic effect of THZ1. At the same time, *TP53* loss did not result in a complete loss of THZ1 activity. THZ1 continued to suppress *MYC* and *E2F* target programs after *TP53* loss.

Our functional data suggest that *TP53* mainly influences the apoptotic response to THZ1 rather than whether AML cells can respond to THZ1 at all. THZ1 induced p53 accumulation in wild-type MOLM-13 cells, and activation of the p53 pathway with idasanutlin further enhanced its anti-leukemic effect. From a clinical perspective, these findings suggest that pharmacologic activation of p53 may be a rational strategy to enhance the apoptotic component of THZ1 response in *TP53*-intact AML. However, this combination strategy will require further evaluation of genotype-specific efficacy, optimal dosing schedules, and potential toxicity before clinical translation. By contrast, *TP53* loss reduced the sensitivity of AML cells to THZ1-induced apoptosis at each time point examined, but THZ1 still induced progressive cell death over time in *TP53*-knockout cells. Moreover, the IC50 values at 24 h were similar between vector control and *TP53*-knockout cells. Taken together, these observations suggest that *TP53* enhances the apoptotic component of the THZ1 response, but is not essential for THZ1 responsiveness.

To understand why THZ1 remained active after *TP53* loss, we analyzed the transcriptional consequences of *TP53* knockout. *TP53* knockout markedly reshaped the transcriptional state of MOLM-13 cells and was associated with downregulation of *MYC* and *E2F* target programs, DNA repair, G2M checkpoint, and several metabolic signatures. *TP53* knockout was also associated with activation of pathways that may support adaptive survival. Among the upregulated pathways, TNFα/NF-κB signaling and JAK/STAT-related programs are particularly relevant because both have been linked to AML cell survival, poor prognosis, and therapy resistance [30,31]. The enrichment of mTORC1 and hypoxia-related programs further suggests stress-adaptive remodeling, as these pathways are involved in AML cell survival, chemoresistance, and microenvironmental protection [32,33]. These resistance-associated programs may help cells with *TP53* loss tolerate therapeutic stress and may contribute to the reduced apoptotic response to THZ1. Notably, when the *TP53*-knockout transcriptome was compared with the CRISPR screen, *MYC* and *E2F* target programs were the only pathways shared by both analyses. Thus, although these programs were reduced after *TP53* loss, they remained the most consistent transcriptional features linked to THZ1 response. Subsequent validation supported this interpretation. In *TP53*-knockout MOLM-13 cells, THZ1 still suppressed multiple *MYC* and *E2F* target genes. In THP-1 cells, which harbor mutant *TP53*, THZ1 likewise induced coordinated downregulation of genes from the same *MYC* and *E2F* target programs. These findings suggest that THZ1 continues to suppress *MYC* and *E2F* target programs even when *TP53* signaling is impaired.

These observations are broadly consistent with previous studies of CDK7 inhibition. Prior work has shown that activation of the p53 transcriptional program can increase sensitivity to CDK7 inhibitors, and enhanced p53 expression can further increase THZ1 sensitivity in wild-type p53 settings [16,34]. Conversely, other studies have shown that CDK7 inhibition can remain effective in p53-mutant tumors [35,36]. Mechanistically, this partial dependence on *TP53* may reflect a link between transcriptional perturbation and p53-mediated apoptosis. Consistent with previous studies and our pathway-level analyses, CDK7 inhibition can impair RNA polymerase II-dependent transcription and suppress transcriptional programs related to *MYC* and *E2F* signaling, cell cycle progression, and DNA repair [37,38,39,40,41]. Disruption of these transcriptional and proliferative programs may create cellular stress that activates or amplifies p53-associated apoptotic responses in *TP53*-intact cells [42]. When *TP53* is lost, this p53-associated apoptotic amplification would be attenuated, which may explain the reduced THZ1-induced apoptosis observed in *TP53*-knockout cells. However, *TP53* loss did not prevent THZ1 from inhibiting CDK7-regulated transcriptional programs. Thus, AML cells may still experience suppression of transcriptional programs that support leukemic proliferation and survival, including *MYC* and *E2F* target programs. This retained transcriptional suppression may help explain why THZ1 activity was attenuated but not abolished after *TP53* loss. The precise mechanisms linking these transcriptional changes to *TP53*-independent cell death require further investigation.

Overall, our study shows that *TP53* modulates the anti-leukemic response to THZ1 in AML, but does not fully determine it. Although p53 signaling contributes to THZ1-induced apoptosis, THZ1 continues to suppress *MYC* and *E2F* target programs in *TP53*-knockout MOLM-13 cells and *TP53*-mutant THP-1 cells.

Several limitations of this study should be acknowledged. Our mechanistic analyses were mainly based on AML cell line models, and future studies using primary AML samples and patient-derived xenograft models with different genetic backgrounds, including different *TP53* statuses, will be important to validate these findings in more clinically relevant settings. Although *TP53* was functionally validated as the top positively selected hit, other candidate hits, particularly negatively selected genes such as *HEATR1*, were not individually validated and require further validation. Baseline proliferation and matched DMSO-treated controls were not systematically assessed in the time-course apoptosis assay, and direct comparisons of residual viable-cell fractions between vector control and *TP53*-knockout cells should therefore be interpreted with caution. In addition, although *MYC* and *E2F* target programs were the most consistent signals associated with THZ1 response, their precise contribution to the retained activity of THZ1 after *TP53* loss remains to be defined.

## Data Availability

The raw sequence data reported in this paper have been deposited in the Genome Sequence Archive in National Genomics Data Center, China National Center for Bioinformation/Beijing Institute of Genomics, Chinese Academy of Sciences (GSA-Human: HRA013870) that are publicly accessible at https://ngdc.cncb.ac.cn/gsa-human, accessed on 10 May 2026. The remaining datasets used and analyzed during the current study are available from the corresponding author upon reasonable request.

## Supplementary Materials

The following supporting information can be downloaded at https://www.mdpi.com/article/10.3390/biomedicines14051113/s1, Table S1: Primer sequences used in this study.
